# Predictive Value of Ultrasound-Measured Quadriceps Depth and Frailty Status for Hypotension in Older Patients Undergoing Reverse Total Shoulder Arthroplasty in the Beach Chair Position under General Anesthesia

**DOI:** 10.3390/jpm14060642

**Published:** 2024-06-16

**Authors:** Sang-Mee An, Hyun Jung Lee, Jae Hee Woo, Ji Seon Chae, Sang-jin Shin

**Affiliations:** 1 Department of Anesthesiology and Pain Medicine, College of Medicine, Ewha Womans University, Seoul 07804, Republic of Korea; 2 Department of Orthopedic Surgery, College of Medicine, Ewha Womans University, Seoul 07804, Republic of Korea

**Keywords:** sonography, quadriceps depth, sarcopenia, hypotension, shoulder surgery, beach chair position

## Abstract

The beach chair position (BCP) is widely used in shoulder surgery; however, it frequently leads to hypotension. Hypotension in BCP is prevalent among older patients who are at risk of secondary complications such as ischemic injuries. Therefore, this prospective study aimed to investigate the association and predictive value of frailty, as assessed by ultrasound-measured quadriceps depth and questionnaire, in patients aged ≥65 years undergoing elective shoulder surgery under general anesthesia. A multivariable logistic regression analysis was performed to identify independent risk factors for hypotension in BCP under general anesthesia. Receiver operating characteristic curves were constructed to assess the predictive values of various parameters. The results indicated that a quadriceps depth < 2.3 cm and BCP for an extended period significantly increased the risk of hypotension. The combined consideration of quadriceps depth < 2.3 cm and frailty demonstrated markedly superior predictive power compared with each factor individually. In conclusion, the study findings facilitate the screening and identification of risk factors for older patients undergoing surgery in BCP, thereby enhancing perioperative management.

## 1. Introduction

With the increase in the aging population, the prevalence of degenerative shoulder diseases has also been on the rise, contributing to the increasing number of shoulder surgeries among older individuals. For certain shoulder diseases, such as rotator cuff tears, rheumatoid arthritis, and proximal humeral fractures, surgical treatment is often preferred owing to its superior clinical outcomes [1]. Shoulder surgery is commonly conducted with the patient in the beach chair position (BCP). The major advantages of this position include enhanced access to the shoulder joint, minimized bleeding, and a better intra-articular anatomical view [2,3]. However, moving a surgical patient from the supine position to BCP under general anesthesia can lead to hypotension, which may cause serious complications, such as cerebral desaturation, hypotensive ischemia, and neurologic injuries [4,5]. The prevalence of hypotension following the change in position to BCP reaches as high as 50%, making it a major concern for anesthesiologists [4]. Furthermore, hypotension in BCP tends to occur more frequently in older patients, for whom recovering from the adverse effects of hypotension can be more challenging and may be associated with potentially fatal outcomes [3].

Frailty, which is defined as a diminished ability to recover from a physiologically stressful event, is a reported risk factor for perioperative complications in older patients [6]. We recently reported that frailty is associated with the incidence of intraoperative hypotension in older patients undergoing non-cardiac surgeries [7,8]. Therefore, it is reasonable to consider frailty as a risk factor for hypotension in older patients undergoing shoulder surgery in BCP, although the evidence regarding the association between hypotension and frailty is insufficient.

Recent studies have attempted to expand the assessment tools for frailty by incorporating questionnaire-based evaluations and imaging findings, such as quadriceps depth and cross-sectional area of the rectus femoris measured by ultrasound and cross-sectional area of the psoas muscle measured by computed tomography [8,9]. Ultrasound is widely available, easy to use, and safe as it uses nonionizing radiation. We hypothesized that hypotension in BCP would be more prevalent among frail patients and could be identified using screening tools, such as ultrasound and questionnaire assessment. Therefore, we conducted a prospective observational study to investigate the association between frailty, as assessed using a questionnaire and ultrasonographical measurement of quadriceps depth, and hypotension induced by BCP under general anesthesia and to compare their predictive power in detecting hypotension in BCP.

## 2. Materials and Methods

### 2.1. Study Population

Patients aged ≥65 years who underwent elective reverse total shoulder arthroplasty (RTSA) in BCP under general anesthesia were enrolled. Patients with arrhythmias, pulmonary hypertension, implanted pacemakers, angiotensin-converting enzyme inhibitors, angiotensin II receptor blockers, or preoperative hypotension (blood pressure < 65 mmHg) were excluded from the study.

### 2.2. Anesthetic Procedure

All patients received a single-shot interscalene brachial plexus block (ISB), administered by an experienced anesthesiologist (H.J.L.) for postoperative pain control and supplemental intraoperative analgesia. The injection was administered upon arrival in the operating room before the initiation of general anesthesia. Midazolam (3 mg) and fentanyl (50 μg) were used for sedation prior to ISB, and 12 mL of 0.5% ropivacaine with 5 mg dexamethasone was injected under ultrasound-guidance. General anesthesia was induced using glycopyrrolate (0.2 mg), propofol (2 mg × kg^−1^), fentanyl (1–2 μg × kg^−1^), and rocuronium (0.6 mg × kg^−1^), followed by tracheal intubation. Anesthesia was maintained using 50% oxygen in air and desflurane, with a bispectral index of 40–60, and titrated based on the hemodynamic response of the patient. Invasive arterial blood pressure was measured by radial arterial cannulation of the non-operative arm, with the pressure transducer zeroed at the external auditory meatus. The patients were positioned with the back portion of the table angled at 40–45°, with the hip and knee flexed, and remained in this position throughout the operation. All operations were performed by the same senior surgeon. At our hospital, all antihypertensive drugs except diuretics are administered with a sip of water on the day of the operation.

### 2.3. Hypotension in BCP

Hypotension was defined as a mean blood pressure < 65 mmHg, or 20% below the baseline pressure. Such types of hypotension have previously been associated with adverse postoperative outcomes, such as acute kidney injury and myocardial infarction [10,11]. The incidence of hypotension during the first 20 min of BCP was recorded. Patients were categorized into two groups according to the development of hypotension in the relevant time frame; those who experienced hypotension after adopting BCP were classified as the hypotension group, and the remainder as the non-hypotension group. If hypotension occurred after induction of general anesthesia, appropriate medication or fluid loading were administered to normalize blood pressure before changing the position to BCP. The hypotension induced by BCP was treated with 5 mg ephedrine, a fluid bolus, or both, at the discretion of the attending anesthesiologist.

### 2.4. Measurements

#### 2.4.1. Frailty: Reported Edmonton Frail Scale (REFS)

The REFS conceptualizes aging as the accrual of deficits and regards frailty as a multidimensional risk state that can be quantified by the number of deficits rather than as a single characteristic or health problem [12]. The REFS examines nine domains: cognition, health status, functional dependence, social support, medication, nutrition, mood, continence, and functional performance. Each positive response within a domain has a score of 1 or 2 points, which are added to obtain the maximum REFS score (17 for non-hip operations and 18 for hip operations).

Higher scores denote greater frailty. We defined “non-frail” as a score ≤ 5, “prefrail” as a score of 6–7, and “frail” as a score ≥ 8. REFS scores were measured preoperatively at the presurgical center, on the day before or day of surgery.

#### 2.4.2. Ultrasound Image Acquisition and Measurement of Quadriceps Depth

**Figure 1:**
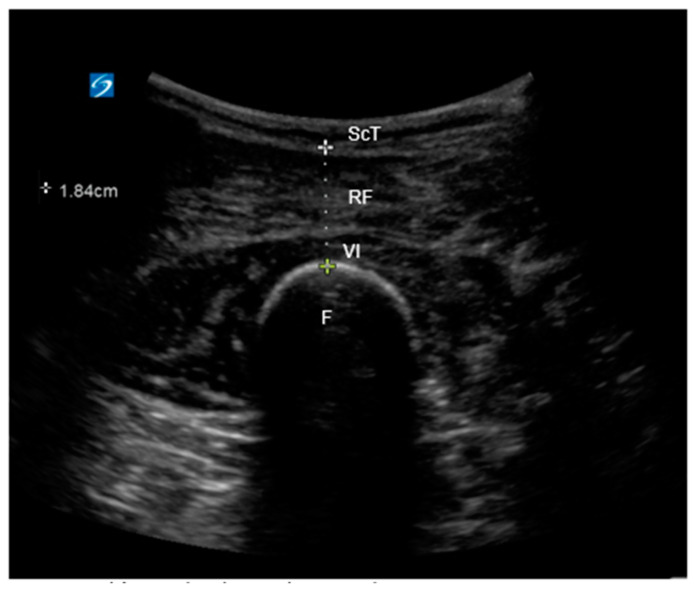
Quadriceps depth on an ultrasound image. The quadriceps depth was obtained by measuring the distance between the cortex of the femur and the most superficial muscular fascia. ScT = subcutaneous tissue; RF = rectus femoris; VI = vastus intermedius; F = femur.

Two-dimensional ultrasound images of the quadriceps muscle were obtained with the patients in the supine position, with 30° upper body elevation and legs extended. The measurements were taken at the 60% mark along the length from the anterior superior iliac spine to the superior border of the patella. In ultrasonography, following the identification of the muscle tissue, quadriceps depth was determined by measuring the distance between the femur’s cortex and the outermost muscular fascia (Figure 1). Three consecutive images of the right and left legs were acquired for each patient using a Sonosite X-porte ultrasound system (FUJIFIML Sonosite, Bothell, WA, USA) and a curved-array transducer with 5–2-MHz bandwidths and were then averaged. This assessment was performed as the initial step upon the patient’s admission to the operating room. All images were acquired, and all measurements were performed by the same trained and certified sonographer, who was blinded to the patient’s frailty status. In an effort to standardize mass composition across body types (e.g., variations in muscle ratio between men and women or differences in muscle quantity between obese patients with high versus low muscle mass), values were indexed by dividing with the body surface area (BSA) and body mass index (BMI) for normalization [9]. We adopted a cut-off value of <2.3 cm for the quadriceps depth based on a previous study to discriminate between frail and non-frail patients [9].

### 2.5. Statistical Analysis

The primary outcome was the prediction of hypotension following position change to BCP, based on frailty evaluation using both “REFS score” and “quadriceps depth measurement by ultrasound.” Based on previous studies, an area under the curve (AUC) defined as the predictive ability of ultrasound-measured quadriceps depth to detect hypotension was assumed to be 0.8, and the probability of being frail in the group receiving RTSA was assumed to be 19.9% [9]. Thus, the calculated sample size was 45 patients (α = 0.05, power = 80%), and accounting for a dropout rate of 10% required 50 patients. Continuous variables were analyzed using the Mann–Whitney U test or Student’s *t*-test after assessing the normality and are presented as means (standard deviation) or medians (interquartile range), as appropriate. Univariate analyses were first performed to explore the associations between variables of interest and the occurrence of hypotension in BCP under general anesthesia. Subsequently, a forward stepwise multivariable logistic regression analysis was conducted to identify the independent risk factors for hypotension in BCP under general anesthesia. All covariates with a *p*-value ≤ 0.2 in the univariable analysis were included in the multivariable logistic regression analysis. The results are expressed as odds ratios (ORs) with 95% confidence intervals (CIs). Receiver operating characteristic curves were constructed to explore the sensitivity and specificity of each identified independent predictor for hypotension, and AUC values were calculated. Statistical significance was set at *p* < 0.05. All statistical analyses were performed using IBM SPSS Statistics (ver. 26.0; IBM Corp., Armonk, NY, USA).

## 3. Results

### 3.1. Study Population

We enrolled 50 patients aged ≥65 years who were scheduled to undergo RTSA. Among them, 4 patients were excluded because 3 patients had their surgery canceled and 1 patient withdrew informed consent, resulting in 46 patients being finally enrolled. The median age of the participants was 72.5 years. Patients were categorized into non-frail, prefrail, or frail groups based on the REFS scores.

### 3.2. Clinical Characteristics of Patients Experiencing BCP-Induced Hypotension

BCP-induced hypotension occurred in 67.4% of patients, including 61.3% of non-frail, 71.4% of prefrail, and 87.5% of frail patients (*p* = 0.462). Additionally, there were no occurrences of long-term complications, such as delirium, ischemic disease, or postoperative cognitive dysfunction, during post-surgery hospitalization. The directly measured quadriceps depth exhibited a significant difference according to the frailty status: 2.04 ± 0.57 (cm) in the non-frail group; 1.82 ± 0.41 (cm) in the prefrail group; and 1.56 ± 0.60 (cm) in the frail group (*p* = 0.041). This difference remained consistent even after normalization by BMI (*p* = 0.040). However, when normalized by BSA, this consistency was not observed (*p* = 0.093). The differences in characteristics between the hypotension and non-hypotension groups are shown in Table 1.

### 3.3. Risk Factors for Hypotension Induced by BCP

Univariate analysis revealed that female sex (reference: male), low quadriceps depth (quadriceps depth < 2.3 cm), duration of anesthesia, duration of surgery, and BCP were associated with hypotension in BCP (Table 2). In the multivariable logistic regression analysis, quadriceps depth < 2.3 cm was a strong risk factor for hypotension in BCP (OR, 8.491; 95% CI 1.389–51.897; *p* = 0.011; Table 2). In addition, the duration of BCP increased the odds of developing hypotension (OR, 1.343; 95% CI 1.102–1.734; *p* = 0.029; Table 2). As the duration of BCP was correlated with the duration of surgery, only the duration of BCP was included as a variable in the multivariate analysis. Results of the multicollinearity test yielded a variance inflation factor < 2.0 for variables in multivariate logistic regression analysis.

### 3.4. Predictors of Hypotension during BCP

**Figure 2:**
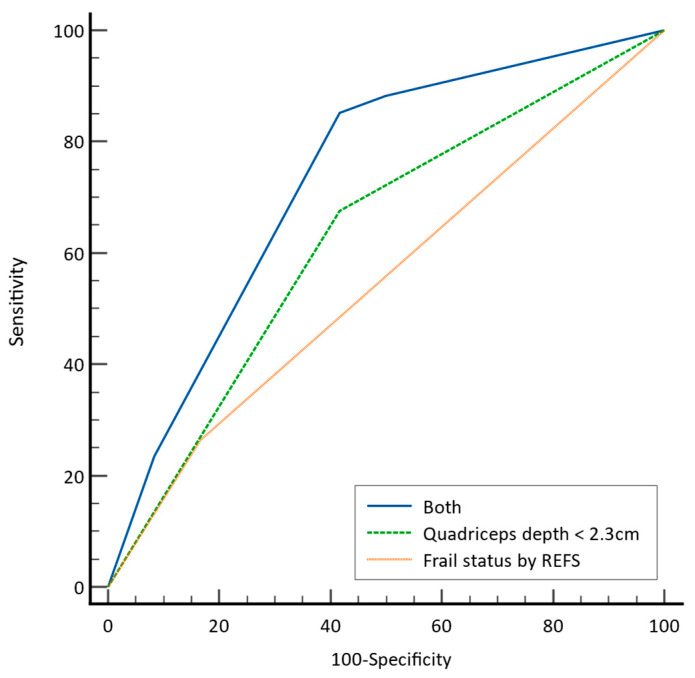
Comparison of the predictive power of the three factors for hypotension during BCP. The three factors are quadriceps depth < 2.3; frail status assessed by REFS scores; and both factors combined. BCP = beach chair position; REFS = Reported Edmonton Frail Scale.

We compared three factors as predictors of hypotension during BCP: quadriceps depth < 2.3 cm, which was reported as a significant imaging criterion for evaluating frailty; frail status based on questionnaire assessment (REFS) scores; and the combination of both factors. The predictive power of the combination of both factors was significantly higher than that of each factor alone (AUC [95% CI]; both, 0.734 [0.583–0.853]; quadriceps depth < 2.3 cm only, 0.630 [0.475–0.767]; REFS-based frail status only, 0.549 [0.395–0.696]) (Figure 2).

## 4. Discussion

This study demonstrated that clinically significant hypotension requiring pharmacologic intervention occurs frequently in approximately 67.4% of older patients undergoing RTSA in BCP under general anesthesia. Besides, hypotension was more commonly observed in frail patients, and frailty appeared to increase the risk of hypotension. A low quadriceps depth (<2.3 cm) was also associated with an increased risk of hypotension in BCP, especially when maintaining the position for an extended period in older patients. While a low quadriceps depth or frail status alone may not predict hypotension in BCP, combining both factors may help in predicting hypotension in BCP.

Severe ischemic brain or spinal cord injury, such as stroke, coma, and quadriplegia, has been reported to occur secondary to hypotension in patients undergoing shoulder surgery in BCP [4]. Although the incidence rate of neurological injury is numerically low [13], when it does occur, it is difficult to recover from such an injury. The prevalence of hypotension during general anesthesia in BCP is reportedly approximately 50% [14,15]. In this study, the incidence of hypotension was as high as 67.4% overall, with an incidence of 87.5% in frail patients. When considering a previous study that reported aging as a risk factor for hypotension induced by BCP, the increased incidence in our study compared to previous studies may be attributed to our study population, which targeted individuals aged ≥65 years [3].

Hypotension associated with BCP is mainly caused by a reduction in the cardiac preload during general anesthesia [16]. In the sitting position, venous pooling in the lower extremities decreases central blood volume [17]. Although blood pressure is restored through compensatory mechanisms, general anesthesia suppresses these actions, making the individual more vulnerable to low blood pressure. Hypotension in the BCP and orthostatic hypotension may share a similar triggering mechanism and engage the corresponding efferent reflex pathway [14]. Additionally, orthostatic hypotension has been reported to be poorly tolerated in frail patients [18,19,20]. Thus, several pathologies associated with frailty may contribute to the occurrence of hypotension in BCP [21,22]. First, frailty has been related to impaired autonomic cardiovascular control, which increases the prevalence of hypotension in BCP [23]. Moreover, age-related factors commonly observed in older patients, such as diminished α-1 adrenergic vasoconstrictor responses to sympathetic stimuli and heightened vascular stuffiness, pose challenges for frail older patients in maintaining their blood pressure in BCP. Second, decreased muscle mass may contribute to the development of hypotension in BCP owing to the impaired homeostatic ability to maintain blood pressure by effective venous return, which is exacerbated by general anesthetic agents [22,24]. Indirect support for this notion could be found in studies suggesting that low muscle mass may elevate the risk of developing orthostatic hypotension through mechanisms involving increased insulin resistance, inflammation, and arterial stiffness [25]. Additionally, the renin-angiotensin system is known to affect the skeletal muscle through complex biochemical pathways and impaired insulin resistance [26]. The fluctuations in blood pressure affect blood flow in muscle, proteins involved in muscle apoptosis, and the synthesis and degradation of biochemical mediators including extracellular matrix, connective tissue growth factor, transforming growth factor-beta, and Glut-4. The interactions of all these biochemical factors are significant in regulating muscle atrophy, muscle perfusion, and muscle fibrosis. This knowledge suggests a relationship, as indicated by our study results, where older patients with low muscle mass exhibited unstable blood pressure variations [26]. Furthermore, a recent study reported the impairment of muscle sympathetic nerve activity owing to reduced muscle mass, leading to orthostatic hypotension [27]. Although no specific criterion for muscle-mass reduction based on ultrasound evaluation has been established yet [28], we found that quadriceps depth < 2.3 cm increased the risk of hypotension in the upright position, indicating that muscle-mass reduction could increase the risk of hypotension in BCP. Third, inflammation, which is a well-known cause of frailty and potentially induced by muscle-mass reduction, could disrupt blood pressure regulation [22]. In our study, no patient had abnormally high levels of inflammation markers such as C-reactive protein, erythrocyte sedimentation rate, or procalcitonin. This may be because our study only included patients undergoing elective surgery. It is possible that surgery was postponed for patients with excessively high inflammation markers; thus, they were not included in our study population.

Although the incidence of hypotension in BCP is notably high, only two studies have explored its predictive indicators to date. Jo et al. [5] suggested that cardiac performance-related hemodynamic values assessed in the pre-induction period could predict hypotension in BCP. However, their study targeted only healthy patients under 65 years of age. Choi et al. [3] included patients of all age groups beyond 18 years of age in determining the risk factors for symptomatic hypotensive bradycardia events occurring in BCP and identified aging as a strong risk factor. To the best of our knowledge, the identification of risk factors in the vulnerable older population prone to hypotension, along with the determination of predictive indicators, represent novel and distinctive aspects of our study that underscore its strength.

Preoperative fasting-induced hypovolemia is recognized to potentially precipitate hypotension during anesthesia, with vasodilation induced by general anesthetics; BCP may exacerbate this phenomenon. Therefore, the patient’s volume status is likely to significantly influence the occurrence of hypotension. Although this study did not include information on the patients’ volume status, such as their stroke volume responses to fluid challenges, we observed no significant difference in the nil per os (NPO) duration between the non-hypotension and hypotension groups. Our hospital adheres to a preoperative regimen where the patients fast from midnight, abstain from carbohydrate beverages, and receive NPO fluids (such as Hartman solution or plasma solution A) at a gradual infusion rate of either 40 or 60 mL/h starting at 5:00 a.m. on the day of surgery. Therefore, we consider that the lack of preoperative volume status measurements does not detract from our findings, which are that preoperative frailty and quadriceps depth measurement are associated with hypotension during BCP.

However, this study has some limitations. First, because our research primarily focused on the occurrence of hypotension, we did not monitor the regional cerebral tissue oxygen saturation. Therefore, we were unable to explore the relationship between preoperative frailty or quadriceps depth and cerebral oxygen desaturation events, despite the potential significance of such events as secondary outcomes of hypotension. Second, we did not assess the severity or duration of hypotension, nor did we include an analysis of the recovery time from hypotension. Consequently, we were unable to conduct a more diverse analysis regarding the influence of preoperative frailty or quadriceps depth on the incidence of hypotension in BCP. Third, our study participants received only desflurane, which can increase the heart rate, as the maintenance anesthetic [29]. Although the incidence of hypotension in BCP is reportedly lower in the higher heart-rate group, we administered desflurane exclusively to all patient groups [5]. Therefore, further research is needed to investigate the influence of different anesthetics. Fourth, to ensure a more accurate evaluation, future studies should incorporate functional assessments, such as hand-grip strength testing, along with measurements of muscle mass. Our study only assessed limited functional capabilities, such as walking, during the questionnaire evaluation. Therefore, although sarcopenia can be suspected based on quadriceps depth, a definitive diagnosis requires the inclusion of functional assessment components. This comprehensive approach would provide a clearer understanding of the risk factors for hypotension resulting from BCP.

In conclusion, this study demonstrates the association between preoperative frailty and quadriceps depth and hypotension during BCP. Considering both of these factors together enhances the prediction of hypotension events in BCP; thus, healthcare professionals should consider the possibility of hypotension in BCP when evaluating older frail patients with suspected sarcopenia.

## Figures and Tables

**Table 1:** Comparison of demographics and intraoperative characteristics between the non-hypotension group and hypotension group during BCP.

	Non-Hypotension Group (*n* = 15)	Hypotension Group (*n* = 31)	*p*
Age (years)	72.3 (67.0–77.25)	72.5 (66.2–74.0)	0.233
Sex (% of males)	66.7	38.7	0.116
ASA physical status classification			0.571
1	1	4	
2	11	24	
3	3	3	
Frailty (REFS scores)			0.462
Non-frail (0–5)	12	19	
Prefrail (6–7)	2	5	
Frail (8–18)	1	7	
Depth of quadriceps (cm)	2.42 ± 0.48	1.92 ± 0.55	0.007
Normalized quadriceps depth by BMI	0.093 ± 0.17	0.075 ± 0.021	0.012
Normalized quadriceps depth by BSA	1.396 ± 0.215	1.165 ± 0.324	0.027
BMI (kg/m^2^)	26.1 ± 1.64	25.63 ± 30.4	0.508
BSA (m^2^)	1.73 ± 1.57	1.65 ± 1.73	0.172
Hypertension history (n)	7	25	0.467
Diabetes mellitus history (n)	3	7	0.706
Induction dosage of propofol (mg/kg)	1.36 ± 0.38	1.27 ± 0.19	0.548
Dosage of ephedrine used before BCP			
Maximum desflurane concentration (%)	4.17 ± 0.94	3.90 ± 0.90	0.383
Duration of anesthesia (min)	146.25 ± 12.99	152.56 ± 9.61	0.144
Duration of surgery (min)	86.25 ± 10.90	96.03 ± 13.80	0.02
Duration of BCP (min)	96.67 ± 10.94	107.5 ± 14.26	0.012
Angle of operation table during BCP (°)	42.92 ± 1.56	42.56 ± 1.58	0.505
Midnight to start of procedure, hours	9.2 [8.1–10.3]	9.5 [8.3–10.6]	0.834
Estimated blood loss (mL)	50 (40, 57.5)	50 (40, 55)	0.937
Fluid administration during BCP (mL)	758.33 ± 144.34	988.53 ± 246.04	0.004
First onset of hypotension		4.5 (1.0–5.0)	
Number of episodes of hypotension for each patient		2 (1, 3)	
Ephedrine (mg) used during BCP		10 (5, 15)	

Data are presented as numbers, means ± standard deviation, or medians (interquartile range); ASA = American Society of Anesthesiologists; REFS = Reported Edmonton Frail Scale; BCP = beach chair position; BMI = body mass index; BSA = body surface area.

**Table 2:** Variables associated with the incidence of hypotension during BCP.

	Hypotension during BCP	
	Odds Ratio (95% CI)	Adjusted Odds Ratio (95% CI)
Age, years	1.069 (0.955–1.197)	0.973 (0.832–1.139)
Sex (reference: male)	2.927 (0.756–11.337)	1.705 (0.20–11.654)
ASA physical status classification		
1	*Reference*	
2	0.591 (0.059–5.905)	
3	0.750 (0.032–17.506	
Hypertension	1.984 (0.5–7.867)	
Diabetes mellitus	0.778 (0.165–3.660)	
Frailty group (REFS score)		
Non-frail group (0–5)	*Reference*	
Prefrail group (6–7)	1.157 (0.263–9.476)	1.163 (0.342–9.013)
Frail group (8–18)	4.421 (0.932–40.56)	5.732 (1.003–32.24)
Quadriceps depth (cm)	0.172 (0.042–0.705)	0.292 (0.014–6.087)
Normalized quadriceps depth by BMI	0.715 (0.01–30.71)	
Normalized quadriceps depth by BSA	0.818 (0.002–40.78)	
Quadriceps depth < 2.3 cm	6.720 (1.502–30.071)	8.491 (1.389–51.897)
Duration of anesthesia (min)	1.058 (0.991–1.129)	
Maximum desflurane concentration %	0.723 (0.352–1.484)	
Duration of surgery (min)	1.065 (1.003–1.1132)	
Duration of BCP (min)	1.066 (1.006–1.128)	1.343 (1.102–1.734)
Angle of operation table during BCP (°)	0.860 (0.558–1.325)	
Estimated blood loss (mL)	1.018 (0.964–1.076)	

Variables with at *p* < 0.2 were included in the multivariate logistic regression analysis. BCP = beach chair position; ASA = American Society of Anesthesiologists; REFS = Reported Edmonton Frail Scale; BMI = body mass index; BSA = body surface area; CI = confidence interval.

## Data Availability

Data will be made available upon request.
